# Characterization of evolution trajectory and immune profiling of brain metastasis in lung adenocarcinoma

**DOI:** 10.1038/s41698-021-00151-w

**Published:** 2021-02-12

**Authors:** Tao Jiang, Yan Yan, Kun Zhou, Chunxia Su, Shengxiang Ren, Nan Li, Likun Hou, Xianchao Guo, Wei Zhu, Henghui Zhang, Jie Lin, Jun Zhang, Caicun Zhou

**Affiliations:** 1 Department of Medical Oncology, Shanghai Pulmonary Hospital & Thoracic Cancer Institute, Tongji University School of Medicine, 200433 Shanghai, China; 2 Department of Oncology, The First Affiliated Hospital of Zhengzhou University, 450052 Zhengzhou, China; 3 Department of Thoracic Surgery, The First Affiliated Hospital of Zhengzhou University, 450052 Zhengzhou, China; 4 Department of Oncology, The Second Affiliated Hospital of Kunming Medical University, 650101 Kunming, China; 5 Department of Pathology, Shanghai Pulmonary Hospital, Tongji University School of Medicine, 200433 Shanghai, China; 6 Beijing Genecast Biotechnology Co., 100000 Beijing, China; 7 Division of Medical Oncology, Department of Internal Medicine, University of Kansas Medical Center, Kansas City, KS 66160 USA; 8 Department of Cancer Biology, University of Kansas Cancer Center, University of Kansas Medical Center, Kansas City, KS 66160 USA

**Keywords:** Non-small-cell lung cancer, Metastasis

## Abstract

Characterizing the evolutionary trajectory and immune profiling of brain metastasis (BM) may provide insights in the development of novel therapeutic strategies. Here, we performed whole-exome sequencing and multiplex immunofluorescence (MIF) of 40 samples from 12 lung adenocarcinoma (LUAD) patients with BM and compared to their paired primary tumors. We observed significantly higher intertumor heterogeneity between paired primary tumors and BMs, with only a median of 8.3% of genetic mutations identified as shared. Phylogenetic analysis revealed that BM-competent clones genetically diverged from their primary tumors at relatively early stage, suggesting that the parallel progression model is dominant. In cases with synchronous lymph node metastasis (LNM), phylogenetic analysis suggested that BM is a later event than LNM. MIF analysis found that BMs exhibited significantly lower CD8^+^ T cell infiltration (*P* = 0.048), and elevated CD4^+^Foxp3^+^ T cell infiltration (*P* = 0.036) and PD-1 expression (*P* = 0.047) in comparison to the matched primary tumors, indicating an immunosuppressive microenvironment in BMs. The current study revealed the discrepancy of mutational landscape as well as tumor immune microenvironment between BM and its primary tumor – such findings shall help us better understand the unique biological features of BM and develop innovative strategies accordingly for our patients with LUAD.

## Introduction

Distant metastasis represents ominous progression of various solid tumors including lung cancer^1^. Accumulating evidence suggested that distant metastasis is an evolutionary process through which tumor cells spread from the primary lesions^2–4^. Recently, several studies compared the primary lesions and matched metastases, and indicated the existence of disparate evolutionary trajectories of metastasis in different types of cancers, as well as metastasis to different organ sites in the same individual^5–7^.

Brain is one of the common metastatic sites for patients with advanced non-small-cell lung cancer (NSCLC)^8,9^. In spite of recent progress in targeted- and immuno- therapies, NSCLC patients with brain metastasis (BM) often have dismal prognosis due to suboptimal therapeutic effect and rapid disease progression. Previous genomic analysis of solid tumors and matched BMs revealed significant genetic heterogeneity between primary lesions and BMs^10^, and the degree of genetic heterogeneity of BMs varied significantly among individuals with NSCLC, breast, and colorectal cancer^11–13^, indicating intertumor heterogeneity of metastatic disease. In fact, Kudo et al. reported reduced T cell and elevated macrophage infiltration in BMs in comparison to the primary lesions^14^. In addition, several publications showed that BM is genetically different from metastasis in either the regional lymph nodes or other extracranial sites^10–13^, suggesting the complexity and particularity of BM. Although a recent study identified several genetic drivers that could promote BM^15^, the distinct mutational and immune features of BM, as well as its phylogenetic relationship with matched primary lesions and lymph nodes metastasis (LNM) remain largely unknown.

In this study, we performed whole-exome sequencing of 40 tissue and blood samples from 12 LUAD patients with BM (four of them had synchronous LNM), to investigate the evolutionary trajectory and mutational landscape of BM. In addition, multiplex immunofluorescence (MIF) was conducted to study the immune biomarkers. We aimed to gain insights of BM through these studies for the development of future therapeutics.

## Results

### The concordance and discrepancy of mutational landscape between matched primary tumors and BMs

**Fig. 1:**
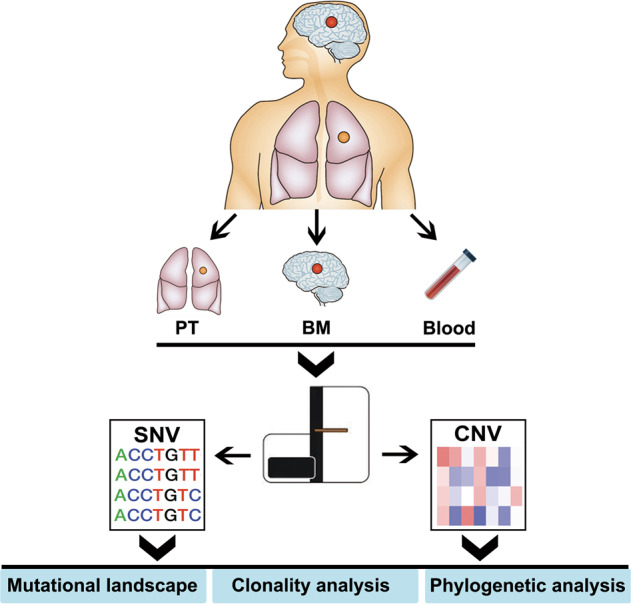
Flowchart of sample collection and experimental procedure. PT primary tumor, BM brain metastasis.

**Fig. 2:**
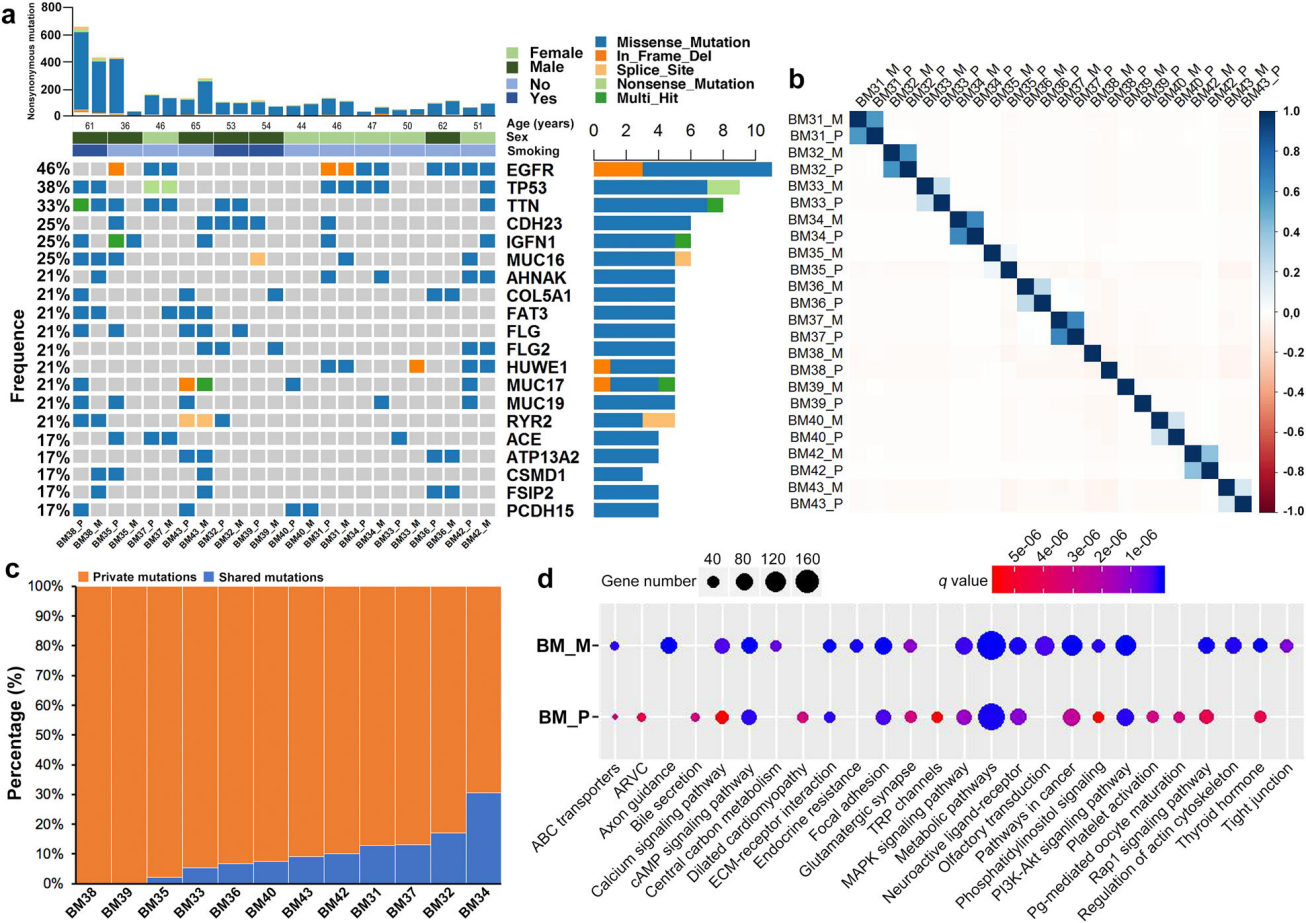
Overview of mutational landscape. **a** Identified mutational landscape of primary lung adenocarcinoma lesions and matched brain metastases (BM). Top panel: the number of somatic mutations in each tumor. Three clinicopathological characteristics (age, sex, and smoking history) are presented below. Left panel: the frequency of listed driver genes. Middle panel: the matrix of mutations in a selection of frequently mutated genes. Columns represent samples. Right panel: the total number of patients harboring mutations in each gene. **b** Pearson Correlation Coefficients of CNVs between primary tumors and BMs in each case. **c** The percentages of shared (present in both primary and metastatic lesions) or private (present only in primary or metastatic lesions) mutations in each case. **d** Top 10 enriched pathways via KEGG pathway analysis in primaries and brain metastases. BM_M brain metastatic lesion, BM_P primary lesion.

We successfully conducted whole-exome sequencing in 40 samples (12 primary tumors, 12 BM lesions, 12 peripheral blood cells, and 4 synchronous LNM lesions) from 12 cases with matched primary lesion and BM (Fig. 1), yielding a median depth of 156× (101–287×). The clinicopathological parameters were summarized in Supplementary Table 1. In total, 6620 unique somatic mutations were identified in all primary (2917 mutations) and metastatic (3703 mutations) tumor tissues by comparing them with the matched peripheral blood samples (Supplementary Fig. 1). Only 10.9% (402/3703) of mutations in BMs were identified in primary tumors. No specific somatic mutations were found to be particularly associated with BM. The most commonly mutated driver alterations were *EGFR* (50.0%, 6/12) and *TP53* (33.3%, 4/12), which were highly consistent between matched primary tumors and BMs (Fig. 2a), indicating that they were predominantly key events. We also noticed 66.7% (4/6) of patients with *EGFR* mutation had concomitant *TP53* alterations.

To infer the underlying mutational processes, the mutation data was categorized into a base substitution matrix and analyzed using the non-negative matrix factorization method implemented in MutationalPatterns(14). In total, four de novo mutational signatures (1–4) were extracted from these samples (corresponding to COSMIC signatures 1–4). Further comparisons was made between these four signatures and the previously defined COSMIC signatures (https://cancer.sanger.ac.uk/cosmic/signatures_v2), which is useful to reveal the clinical relevance of these four signatures. We found the presence of mutational signature 1–4 with signature 2 [apolipoprotein B mRNA–editing enzyme, catalytic polypeptide-like (APOBEC)–associated] as the most dominant (Supplementary Fig. 2A). Most of paired cases showed the heterogeneous proportion of different mutational signatures between primaries and BMs. We did not find obvious difference in the pattern of transition and transversion between primaries and matched BMs (Supplementary Fig. 2A). Majority of these samples displayed a C > T transversions and there was a trend of C > A transitions in patients with smoking history when compared with those without smoking history (Supplementary Fig. 2A). Of note, although primary lesions had higher non-synonymous mutation counts than BMs (309 vs. 244, *P* = 0.265; Supplementary Fig. 2B), it did not reach the statistical significance possibly due to small sample size. There was also no significant difference of Math value between primary lesions and BM (*P* = 0.137; Supplementary Fig. 2C). In addition, we compared the pattern of SNVs and CNVs at the chromosome level between primary tumors and matched BMs using Circos plot. As shown in Supplementary Fig. 3, we observed an obvious discrepancy of mutational landscape but different degree of genetic heterogeneity between matched primary and BM lesions.

To quantitate intertumor heterogeneity between primary and metastatic lesions, we calculated the Pearson Correlation Coefficients (PCC) to evaluate the mutation relatedness between primary tumors and BMs. There was limited relatedness between matched primary tumors and BMs (median PCC, 0.178, range 0.005–0.761; *P* > 0.05; Fig. 2b). We then investigated the “shared” (i.e., genetic alterations including SNVs and CNVs that present in both the primary and metastatic lesions) and “private” (i.e. alterations presenting in either the primary or metastatic lesions) mutations according to previous study^16^. We observed only a median of 8.3% (range, 0.0–30.5%) of genetic mutations were shared, suggesting a high intertumor mutational heterogeneity between paired BM and primary tumors (Fig. 2c).

To further determine whether such mutational discrepancy could result in difference in cancer-related signaling pathways, we performed pathway-level analysis using the Kyoto Encyclopedia of Genes and Genomes (KEGG) database. We listed the top 20 enriched pathways in each group, respectively (Fig. 2d). Comparing to the matched primary tumors, BM had strikingly significant enrichment in multiple pathways. Consistent with previous reports^17–19^, PI3K-AKT signaling pathway was enriched in both primary tumors and BMs (*q* = 8.19 × 10^−8^, *q* = 9.78 × 10^−13^, respectively), which is confirmatory of its universal role in cancer progression^11^. In addition, we observed significant enrichment of invasion/metastasis associated pathways in BMs than their primaries, which included Rap1 signaling pathway (*q* = 0.024), tight junction (*q* = 5.63 × 10^−8^), and regulations of actin cytoskeleton pathways (*q* = 7.23 × 10^−11^). Interestingly, several metabolic pathways such as the calcium signaling (*q* = 0.045), ABC transporters (*q* = 0.013), and central carbon metabolism in cancer pathways (*q* = 5.01 × 10^−6^) were also significantly more enriched in BMs (Fig. 2d).

### Subclonal architecture and phylogenetic relationship between matched primary tumors and BMs

**Fig. 3:**
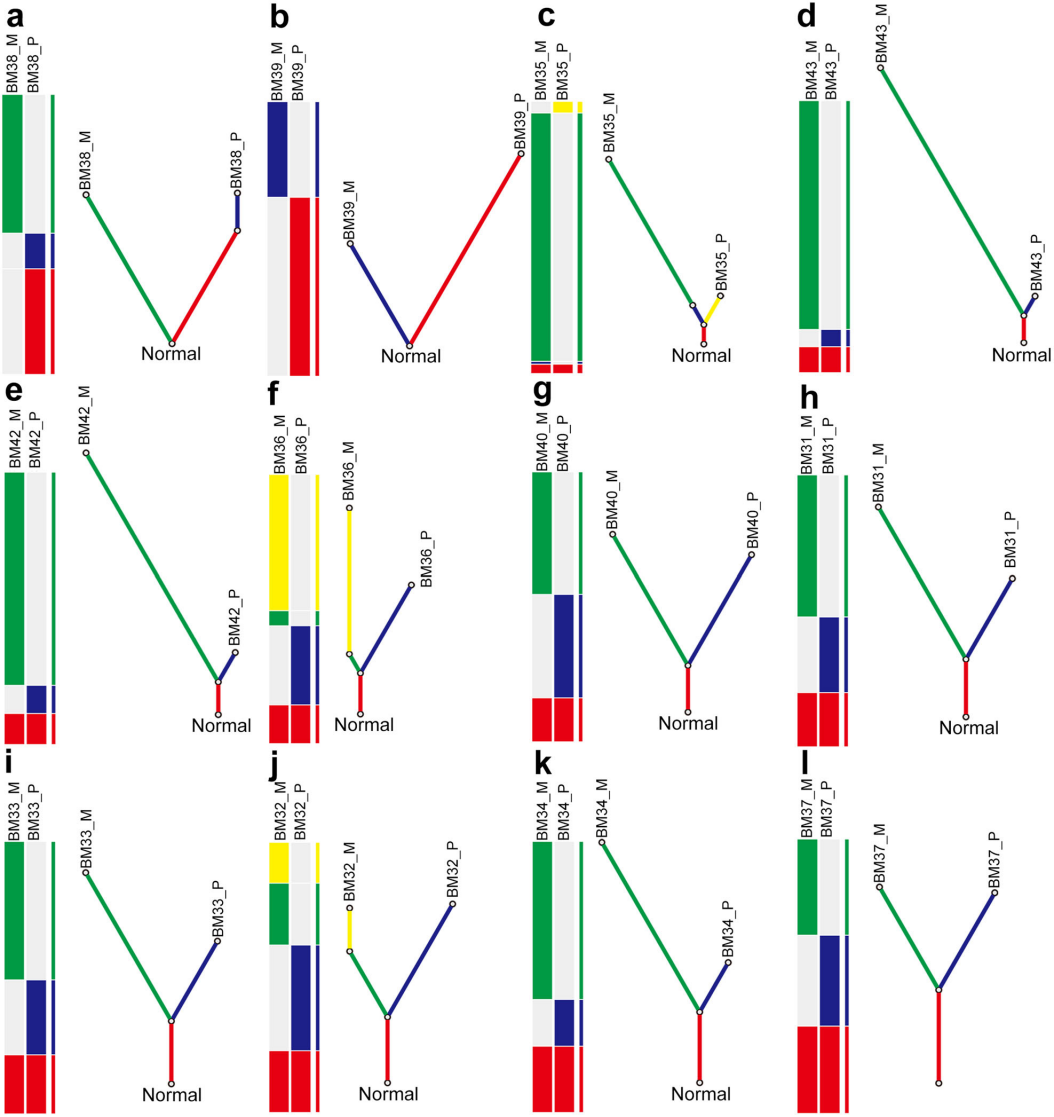
Phylogenetic analysis of cases with brain metastases. **a**–**l** phylogenetic tree of BM38, BM39, BM35, BM43, BM42, BM36, BM40, BM31, BM33, BM32, BM34, and BM37. For each panel, heatplot with different bars represents various distributions of identified mutations including somatic SNVs and CNVs. Fraction of ubiquitous identified mutations (trunk) and unique identified mutations (branch) reveal the phylogenetic relationship of BM_P and BM_M within a single case. BM_P primary lesion, BM_M brain metastatic lesion.

To characterize the evolutionary trajectories of BMs, we first investigated the subclonal architecture of paired tumor samples by using SciClone^5,20,21^. We then inferred the sequence of genomic alterations, followed by reconstruction of phylogenetic tree using LICHeE in each case. The distinct variant allele frequencies (VAFs) of each cluster in primary lesion and matched metastasis suggested variable clonality of different tumors. The subclonal architecture derived from BM showed discrepancy to matched primary lesions and there was limited correlation of the mutation cluster distribution between them (Supplementary Fig. 4). Importantly, we observed that BM-competent clones had higher level of genetic heterogeneity to their paired primary lesions, and genetically diverged from their primary tumors at relatively early stage, suggesting that the parallel progression model is dominant (Fig. 3). Interestingly, we observed that most metastatic lesions (75.0%, 9/12) seemed to have higher counts of somatic mutations than their matched primary lesions (Fig. 3, measured by the length of branches of BM), indicating an accumulation of more mutations under evolution pressure during BM formation.

We have listed two representative cases in Supplementary Fig. 5. BM38 is a 61-years male smoker. Although most identified driver gene mutations were consistent, Circos plot showed the high level of SNV and CNV in BM than in primary tumors (Supplementary Fig. 5A). The subclonal architecture derived from BM38_M showed discrepancy to BM38_P and there was no correlation of the mutation cluster distribution between BM38_P and BM38_M (Supplementary Fig. 5B). Phylogenetic tree showed a very early separation of metastasis-competent clone from primary lesion and there are no shared identified mutations between the primaries and BMs (Supplementary Fig. 5C). Repeat sequencing and analysis of these samples demonstrated this finding and indicated that they are not the case where the patient has developed two independent tumors. BM35 is a young male without smoking history. Circos plot showed highly distinct SNV and CNV pattern between BM35_P and BM35_M (Supplementary Fig. 5D), while identified driver gene mutations were largely discordant (Fig. 2a). Notably, subclonality analysis revealed that some mutation cluster 1 was found in both BM35_P and BM35_M whereas mutation cluster 2 was only found in BM35_P (Supplementary Fig. 5E), suggesting BM-competent clone was mainly originated from mutation cluster 1. Phylogenetic tree confirmed the initial overlap clone and independent evolution and accumulation of mutations during BM formation (Supplementary Fig. 5F).

### Phylogenetic analysis of matched primary tumor, lymph node, and brain metastasis

**Fig. 4:**
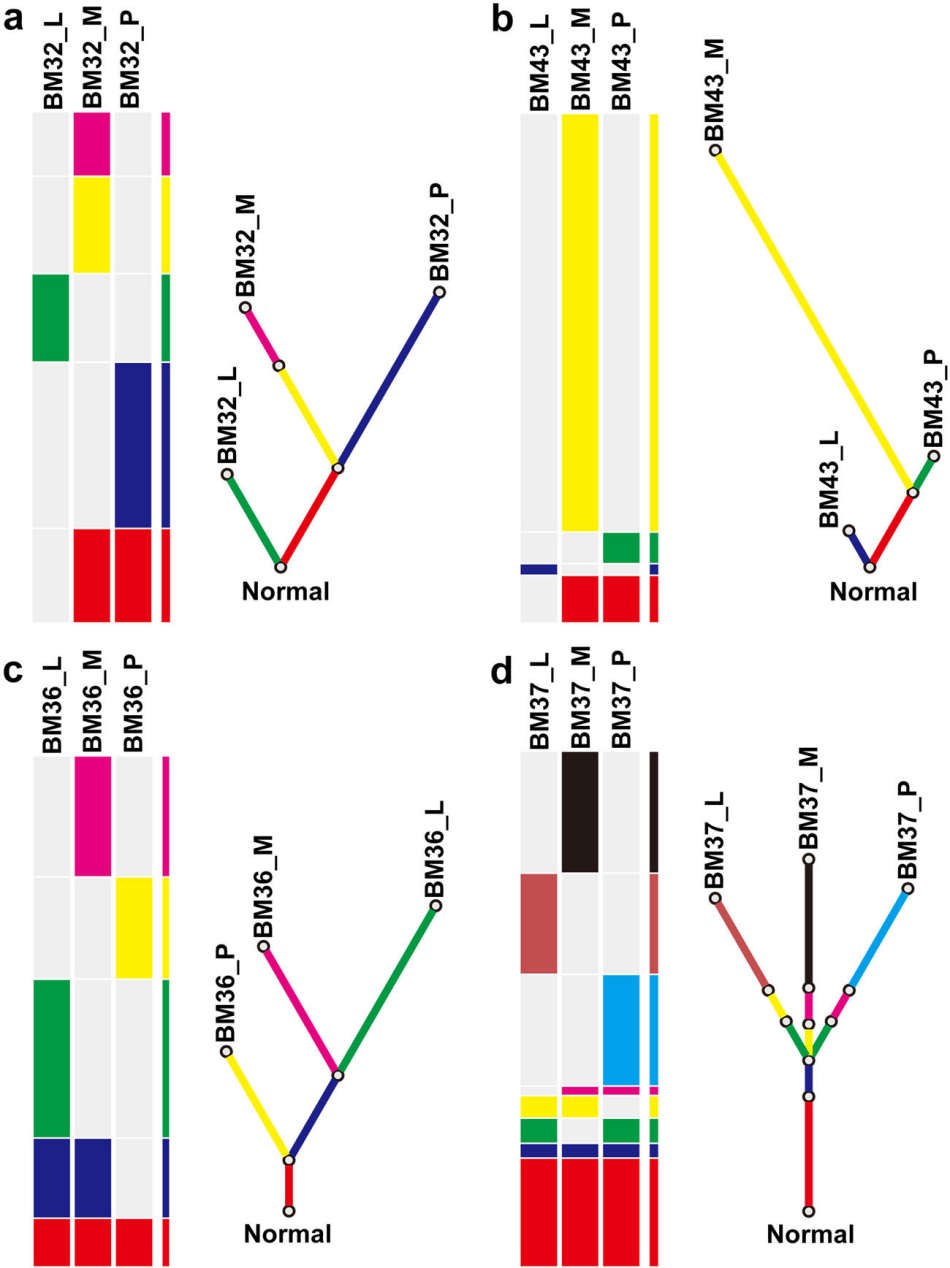
Phylogenetic tree of four cases with synchronous lymphatic and brain metastases. **a**–**d** phylogenetic tree of BM32, BM43, BM36, and BM37. For each panel, heatplot with different bars represents various distributions of identified mutations including somatic SNVs and CNVs. Fraction of ubiquitous identified mutations (trunk) and unique identified mutations (branch) reveal the phylogenetic relationship of BM_P, BM_L and BM_M within a single case. BM_P primary lesion, BM_L lymph node metastasis, BM_M brain metastatic lesion.

In patients with epithelial cancers including lung, colorectal, prostate, and breast cancer^22,23^, LNM is one of the important step before cancer cells spread to pivotal organs^24^. Lymph node involvement often precedes systemic disease and could give rise to distant metastases, which lay the clinical foundation for the TNM staging system^25^. However, whether BM of LUAD share the similar origin to lymphatic metastasis or BM is the subsequent progression of LNM remains undetermined. In our cohort, we collected four cases with synchronous LNM. For BM32 and BM43, BM and primary lesion shared higher degree of clonal similarity than LNM (Fig. 4a, b). This is consistent with BM showed stronger relatedness to the primary lesion in comparing to LNM (Supplementary Fig. 6A, B). In contrast to BM32 and BM43, a higher degree of clonal similarity was observed in BM and LNM of the case BM36 (Fig. 4c), which led to a mutational landscape showing a stronger relatedness of BM to LNM, rather than the primary tumor (Supplementary Fig. 6C). In the case of BM37, after sharing a period of clonality, the BM, LNM, and primary tumor seemed to have individual evolution pattern afterwards based on the phylogenetic tree (Fig. 4d), therefore gave roughly equal genetic relatedness (Supplementary Fig. 6D). Interestingly, despite of such divergent evolution paths, our phylogenetic analysis showed BM-competent clones consistently originated later than LNM-competent clones, therefore is consistent with clinical presentation of cancer progression, and supportive of the application of TNM staging system in our clinical practice.

### Immune profiling between paired primary tumors and BMs

**Fig. 5:**
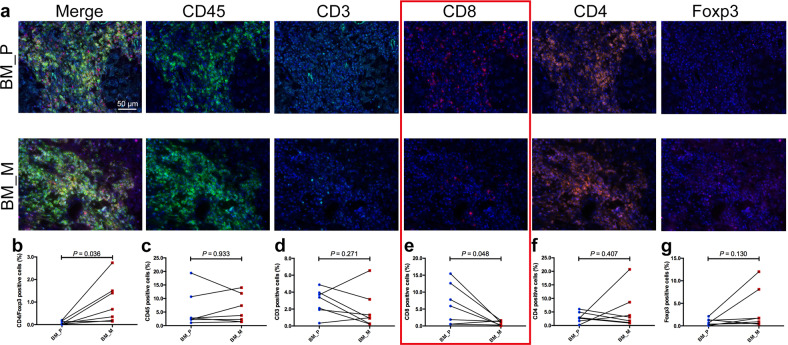
Five immune related markers expression heterogeneity in tumor stroma of primary lesions and BM. **a** Representative images of multiplex immunofluorescence (MIF) of immune related markers including CD45^+^, CD3^+^, CD4^+^, CD8^+^, and Foxp3^+^. MFI comparison of CD4^+^Foxp3^+^ (**b**), CD45^+^ (**c**), CD3^+^ (**d**), CD8^+^ (**e**), CD4^+^ (**f**), and Foxp3^+^ (**g**) cells between primaries and BM. MFI mean fluorescence intensity.

Based on our above-mentioned findings, we further conducted MIF to compare the expression of immune markers between the primary tumors and BMs. We tested CD45^+^, CD3^+^, CD4^+^, CD8^+^, and Foxp3^+^ immune markers in both the tumor core area and tumor-associated stroma. We listed the representative images of each marker in tumor-associated stroma in Fig. 5a. We observed no difference in the MFI of CD45^+^, CD3^+^, CD4^+^, and Foxp3^+^ cells (Fig. 5c, d, f, g) between matched primary tumors and BMs. However, the MFI of CD8^+^ in BMs was found significantly lower (*P* = 0.048; Fig. 5e), and CD4^+^Foxp3^+^ expression was significantly higher (*P* = 0.036; Fig. 5b). Moreover, we observed similar expression pattern of these markers in tumor core area although a statistical significance was not observed, presumably due to relatively small sample size (representative images and MFI of each marker was listed in Supplementary Fig. 7).

**Fig. 6:**
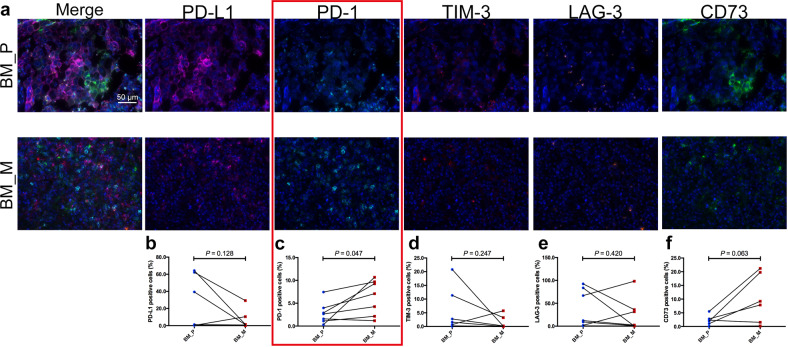
Five immune checkpoints expression heterogeneity in tumor stroma of primary lesions and BM. **a** Representative images of MIF of immune checkpoints expression including PD-1, PD-L1, TIM-3, LAG-3, and CD73. MFI comparison of PD-L1 (**b**), PD-1 (**c**), LAG-3 (**d**), TIM-3 (**e**), and CD73 (**f**) cells between primaries and BM. MFI mean fluorescence intensity.

The expression of five immune checkpoints including PD-1, PD-L1, TIM-3, LAG-3, and CD73 were also tested. The representative images of each immune checkpoint in tumor-associated stroma were listed in Fig. 6a. The MFI of PD-L1, LAG-3, and TIM-3 was comparable between primaries and BMs (Fig. 6b, d, e). However, BMs exhibited markedly higher MFI of PD-1 (*P* = 0.047) than primary tumors (Fig. 6c). Although CD73 MFI was also higher in BMs, it did not reach statistical significance (*P* = 0.063) likely due to limited sample size. In addition, similar expression pattern of these immune checkpoints in tumor core area was observed (representative images and comparison of MFI of each immune checkpoint were shown in Supplementary Fig. 8).

## Discussion

Elucidating the evolutionary pattern and its impact on mutational landscape and immune profiling of BMs may improve the management of this clinical entity. In this study, we sequenced 12 paired lung adenocarcinomas with matched BMs to investigate the evolutionary trajectories and immune profiles. We found although commonly mutated driver alterations were highly consistent between paired primary tumors and BMs, other somatic mutations, mutational signature and pattern were highly distinct. Phylogenetic analysis revealed that BM-competent clones genetically diverged from their primary tumors at early stage, indicating the existence of parallel progression model. Four cases with synchronous LNM showed BM-competent clones consistently originated later than LNM-competent clones, suggesting lymphatic spreading is likely an earlier event than BM, which is consistent with clinical picture of cancer progression. Finally, discrepancy of immune related markers was observed between primary tumors and matched BM. All these results suggest a unique evolutionary trajectory throughout the development of BM.

We firstly investigated the common driver mutations, and found *EGFR* and *TP53* mutations were highly concordant. Consistently, Wang et al. collected 61 NSCLC patients with BM, and found that mutations in major drivers, including *EGFR*, *KRAS*, *TP53*, and *ALK*, were highly concordant between primary tumors and matched BMs (>80%)^11^. Moreover, a recent study performed a pan-cancer analysis of genetic heterogeneity and reported that all driver-gene mutations were homogeneous among metastases from the same primary tumor, indicating that cells within the primary tumors that gave rise to metastases are genetically homogeneous with respect to functional driver-gene mutations. Although our population is enriched for “never smokers”, which explains the high incidence of *EGFR* mutations, the concordance between primary tumors and BMs does suggest the existence of cancer “stemness”.

Having noticed that BM had significantly heterogenous level of SNVs and CNVs than primary lesions, we further looked into their evolution by analyzing their involved cancer-related signaling pathways. Not surprisingly, we found that PI3K-AKT signaling pathway was enriched in both the primary tumors and BMs, which is confirmatory to the universal importance of this pathway^10,11,17,18^. Furthermore, Wang et al.^11^ reported that PI3K-AKT signaling in the primary tumor was associated with shorter BM-free survival. However, whether it is effective to prevent and/or control the BM via targeting PI3K-AKT pathway warrants further research. As expected, the invasion/metastasis associated pathways (e.g. Rap1 signaling pathway, tight junction and regulations of actin cytoskeleton pathways) were highly enriched in BMs than their primaries. Of note, Rap1 signaling pathway plays a key role in cell adhesion and integrin function, and is required for EGFR-mediated metastasis in some tumors^26^. Thus, it is valuable to investigate its significance in BM formation in LUAD. In addition, compared with primary tumors, another important alteration observed were several metabolic pathways including calcium signaling and central carbon metabolism in cancer pathways. Brain is the unique organ with different metabolic environment (e.g. high glucose consumption), which might be the explanation. Therefore, further investigation in metabolomics and targeting cancer metabolism might shed a light to prevent BM.

To systematically depict the cancer evolutionary process, two general progression models, namely “linear progression model” and “parallel progression model”, have been previously proposed^2,27^. These two models are determined by two parameters: (i) the relative timing of the emergence of metastases in the primary lesion; and (ii) the anticipated genetic divergence, characterized by comparing the sum of mutations between the primary lesion and matched metastases^2^. In the linear progression model, the metastasis-competent clone disseminates from the primary tumor late together with limited degree of primary-metastasis genetic divergence. In the parallel progression model, the metastatic clone or subclone arises early in tumorigenesis^27^, and both the primary tumor and the metastasis continue to evolve in parallel, resulting in substantial primary-metastasis genetic divergence. In our study, phylogenetic trees revealed the predominance of parallel progression model in BM. Consistently, Zhao et al.^28^ reported 40 paired primary tumors and metastases including 13 lung cancers, and found that 11 cases followed parallel progression model. These observations raise the necessity to incorporate the genetic information from BM, to gain a better understanding of BM biology and guide tailored therapy for these patients, especially those without driver gene alterations.

Considering the critical and prognostic value of regional LNM in patients with epithelial cancers, previous studies have explored the evolutionary relationship between LNM and distant metastases. For example, Ikram et al.^29^ investigated the role of metastatic axillary lymph nodes and reported that synchronous axillary LNM was not involved in seeding the distant metastasis. A recent study further reported that lymphatic and distant metastases arose from independent subclones in the primary tumor in 65% of colorectal cancer^7^, indicating that a hematogenous route would be common for the formation of distant metastasis, and there could be multiple subclones in the primary tumor that independently seed lymphatic and distant metastases. Interestingly, our results showed that BM-competent clones consistently originated later than LNM-competent clones. Despite of small sample size, such finding is consistent with clinical presentation of disease progression, and the foundation of TNM staging system. Collectively, these findings suggest the complexity of origin and evolution of distant metastases. Better understanding of the molecular evolution of metastases could have implication for the development of effective interventional strategies.

Finally, we performed a direct comparison of immune landscape between primary tumors and BM via testing immune related markers including checkpoints. Despite small sample size, we did observe some obvious differences between paired primary tumors and BMs as previously reported^30^. The ratio of CD8^+^ T cells in BM was lower than those in primary tumor but a higher percentage of CD4^+^Foxp3^+^ cells was found in BM, suggesting a suppressive phenotype of immune microenvironment in BM. Consistently, using the similar approach, a recent study found BM lesions exhibited lower T cell and elevated macrophage infiltration in compare to the primary tumors (*P* < 0.001). These findings together revealed the cold-tumor signature of BM microenvironment^14,31–33^. We also observed a heterogenous expression pattern of other immune checkpoints, especially PD-1 and CD73 expression. This is probably due to the fact that one biopsy from one lesion simply has its limitation, therefore warrants exploration of novel approaches to better recapitulate the whole immune microenvironment of both primary tumors and BMs.

Despite the technologies employed here are not yet in routine practice, our study did convey messages that could be of practical use: (1) never assume the same response in BM due to largely distinct mutational landscape from the primary tumor during the clonal evolutional process – therefore certain BM-directed therapies including radiation should be seriously considered and optimized; (2) BM could represent an immune-suppressive tumor subset, therefore warrants innovative approaches to enhance the recruitment of immune cells to this local microenvironment; (3) Studying BM metabolism could offer another new perspective to tackle this problem.

There are several limitations that should be acknowledged. First, the sample size was small. Thus, the results should be cautiously interpreted, and large-scale investigation is warranted. Second, not all tissue samples were fresh-frozen and some were archival FFPE. FFPE artefacts would affect the findings of evolutionary pattern. To address this issue, we have applied a stringent filtering criteria and observed a high consistency of calculated mutation burdens by using different cutoffs of variant allele frequencies. Third, we did not perform the RNA sequencing to further strengthen our findings. Last but not least, most of the patients were *EGFR*-mutant NSCLC. Hence, it might be difficult to generalize these finding to all NSCLC. However *EGFR* mutation is one of the most common driver gene alterations in NSCLC. Characterizing BM in *EGFR*-mutant NSCLC would have significant implication to other groups (e.g. patients with *ALK*, *ROS1* or *BRAF* alterations).

In summary, our current study characterized the evolution trajectory of BM via its comparison to the matched primary tumor. We have clearly pointed out the discrepancy of mutational landscape as well as tumor immune microenvironment between BM and its primary tumor – such findings shall help us better understand the unique biological features of BM and develop innovative strategies accordingly for our patients with LUAD.

## Methods

### Patients’ selection

Patients with histologically or pathologically diagnosed lung cancer and radiologically (computed tomography and/or magnetic resonance imaging) or pathologically confirmed BM were recruited. Primary lung cancers, matched BM, and peripheral blood (10 mL, EDTA) were collected before any systemic treatment and collection time interval was <1 month between January 2012 and December 2015 in three medical centers (Fig. 1). We also collected synchronous positive LNM samples if the patients received biopsy or surgical resection of them. The study protocol was approved by the Institutional Review Board of Shanghai Pulmonary Hospital, Tongji University School of Medicine (No. 2015-0709). Written informed consent was obtained from all participants. The study was conducted in accordance with the Declaration of Helsinki.

### DNA extraction and library construction

Genomic DNA was extracted from all included samples. The matched peripheral blood leukocytes were utilized as the source for germline DNA control. DNA was extracted from the peripheral blood leukocytes and tumor tissues independently using Qiagen DNeasy Blood and Tissue kit (Qiagen, Hilden, Germany) according to the manufacturer’s instructions. The quality check and library construction were seen in Supplementary Material.

### Whole-exome sequencing

DNA libraries were subjected to whole-exome capture with xGen Exome Research Panel v1.0 (Integrated DNA Technologies), which spans a 39-Mb target region (19,396 genes) of the human genome and covers 51 Mb of end-to-end tiled space. The captured samples were sequenced on an Illumina HiSeq X-TEN platform with a paired-end run of 2 × 150 bp. See also Supplementary Material.

### Data filtering and variant calling

The generated sequencing reads were initially parsed with FLEXBAR for adapter trimming and low quality bases were filtered out^34^. Raw sequence reads were mapped to the human reference genome (hg19) by Burrows-Wheeler Aligner (BWA) aligner v0.7.12^35^. Duplicated reads were then removed from the aligned and sorted BAM files by using Picard 2.2.1. GATK v3.8 was utilized to do local realignment around potential small insertions and deletions (Indels) and base recalibration for next step mutation calling procedures. We used MuTect v1.1.7 to detect single nucleotide variants (SNVs) and Strelka v1.0.14 to call small Indels^36,37^. See also Supplementary Material.

### Mutational signature analysis

Both synonymous and non-synonymous somatic SNVs were analyzed to define mutational signatures including six categories of base substitutions, namely, T > A, T > C, T > G, C > A, C > G, C > T, in each included samples. In view of the 5′ and 3′ flanking nucleotides of a specific mutant base, a total of 96 substitution types exist. We extracted the potential mutational signatures in each sample by using the 30 signatures documented by the COSMIC as reference (R package deconstructSigs)^38^. Then, we analyzed and compared the mean weights of different signatures in both primaries and BMs.

### Copy number profiling

DNA copy number variants (CNVs) were detected by using CNVkit v0.8.5 as we previously described^39^, which compared tumor samples with a pool of all selected patient’s blood cell samples. The processed tab-delimited text file was imported into ‘sequenza’ R package to perform GC-content normalization of the tumor versus normal depth ratio, and allele-specific segmentation. The cellularity, ploidy parameters and copy number profiles were then inferred. Finally, visualization of the data, the model along the genome and the individual chromosomes were generated^40^. See also Supplementary Material.

### Phylogenetic tree construction

To generate the phylogenetic trees from somatic variants, we leveraged a published computational method named LICHeE (Lineage Inference for Cancer Heterogeneity and Evolution)^41^ to reconstruct multi-sample cell lineage trees and infer the subclonal composition of each sample using variant allele frequencies of somatic SNV. The lineage tree of the somatic SNV clusters was built based on the constraint network^41,42^.

### Subclonality analysis

SciClone was used to detect subclonal architecture according to previous reported algorithm^21^. SciClone, a computational method that focused primarily on variants in copy number neutral, loss of heterozygosity (LOH)-free portions of the genome, and identifies the number and genetic composition of subclones by analyzing the VAFs of somatic mutations^21^.

### Multiplex immunofluorescence staining

The slides were deparaffinized in xylene, rehydrated, and washed in tap water before boiling in Tris-EDTA buffer (pH 9; 643901; Klinipath) for epitope retrieval/microwave treatment (MWT). Endogenous peroxidase was blocked using Antibody (Ab) Diluent / Block (72424205, PerkinElmer). After staining with specific antibodies, the stained cells were detected by using inForm Advanced Image Analysis software (inForm 2.3.0; PerkinElmer, Massachusetts, USA). The mean fluorescence intensity (MFI) of each marker expression in each stained cell membrane was quantified as previously described^43,44^. Positive cells were defined as cells with true immunofluorescence signal detected (> median MFI of all stained cells in a given slides) and with right expression pattern. A selection of 5–15 representative original multispectral images was used to train the inForm software (tissue segmentation, cell segmentation, phenotyping tool, and positivity score). All the settings were saved within an algorithm to allow batch analysis of multiple original multispectral images of the same tissue. More than 10 fields per slide were selected to calculate the number, percentage and density of positive cells under the ×200 magnification by two experienced pathologists. The percentage of positive cells in all nucleated cells of the tumor nests and tumor stroma from the selected fields was used for analysis.

### Statistical analysis

All tests were performed with GraphPad Prism v6.0. Comparisons between paired primary tumors and metastases were performed using Student’s *t* test. Wilcoxon signed-rank test and *t* test were run for comparison of copy number variation and mutation frequency between different groups. Mann–Whitney *U*-tests or Kruskal–Wallis rank-sum tests were used for comparisons of continuous variables across groups. To determine differences in enriched pathways between groups, we used two-tailed Fisher’s exact tests with Benjamini–Hochberg correction for multiple hypothesis testing to generate *q* values^45^. Pearson Correlation Coefficients were calculated to evaluate the relatedness of mutations between each pair of samples according to previous publications^46,47^. Spearman’s rank correlation was utilized to assess the correlations between continuous variables. *P* < 0.05 was considered statistically significant.

## Supplementary information

Supplemental Material

reporting-summary

## Data Availability

The data generated and analysed during this study are described in the following data record: 10.6084/m9.figshare.13476732^48^. The whole-exome sequencing data are openly available in the NCBI Sequence Read Archive via the following accession: https://identifiers.org/ncbi/insdc.sra:SRP170084^49^. These data underlie Figs. 2–4 in the related article.
