# Supplementary material for: Characterization of evolution trajectory and immune profiling of brain metastasis in lung adenocarcinoma
Source: NPJ Precis Oncol. 2021 Feb 12;5:6. doi: 10.1038/s41698-021-00151-w (PMC7881241; doi:10.1038/s41698-021-00151-w)
Supplement: Supplementary file 1 — Supplemental Material [file 41698_2021_151_MOESM1_ESM.pdf]

Supplementary Material

Characterization of evolution trajectory and immune profiling of brain metastasis in lung adenocarcinoma

Supplementary Text..**Page 2.**

Supplementary Figure 1...**Page 6.**

Supplementary Figure 2...**Page 8.**

Supplementary Figure 3...**Page 9.**

Supplementary Figure 4...**Page 12.**

Supplementary Figure 5...**Page 14.**

Supplementary Figure 6...**Page 15.**

Supplementary Figure 7...**Page 16.**

Supplementary Figure 8...**Page 17.**

Supplementary Table 1..**Page 18.**

Supplementary Text

Methods

Patients' selection

Surgical specimens and biopsy tissues were snap-frozen in liquid nitrogen within 30 minutes of harvest. Then 8-10 μm fresh frozen or formalin-fixed paraffin-embedded (FFPE) sections were made. Hematoxylin-eosin stained slides were reviewed by experienced pathologists to exam the histological subtype and the proportion of malignant cells relative to nonmalignant stromal cells. The criteria for further sequencing analysis of each sample was that the presence of at least 50% tumor nuclei and less than 20% necrosis presented on histological review of each sample.

DNA extraction and library construction

After DNA extraction, the quality check was conducted using Agilent 2100 Bioanalyzer (Life Technologies, USA) per manufacturer's recommended protocol. Genomic DNA was sheared into 150-200 base pairs (bp) fragments with Covaris M220 Focused-ultrasonicator TM Instrument (Covaris, Massachusetts, USA). Fragmented DNAs were constructed via KAPA Hyper Prep Kit (Illumina platforms, KAPA Biosystems, Massachusetts, USA) according to the manufacturer's protocol. Multiple indexing adaptors were ligated to the ends of the DNA fragments to prepare them for hybridization onto a flow cell. Purification and size selection of the library were performed using AMPure XP magnetic beads (Beckman Coulter, Brea, CA, USA). The concentration and quality of the library was determined using the Qubit 3.0 system (Invitrogen) and Bioanalyzer 2100 (Agilent, Agilent HS DNA Reagent, 5067-4627).

Whole-exome sequencing

DNA libraries were subjected to whole-exome capture with xGen Exome Research Panel v1.0 (Integrated DNA Technologies), which spans a 39 Mb target region

(19,396 genes) of the human genome and covers 51 Mb of end-to-end tiled space. Human Cot-1 DNA (Life Technologies) and xGen universal blocking oligos (Integrated DNA Technologies) were added as blocking reagents to reduce non-specific hybridization. The capture reaction was performed using NimbleGen SeqCap EZ Hybridization and Wash Kit (Roche) and Dynabeads M-270 (Life Technologies) according to manufacturers' protocols. The captured samples were sequenced on an Illumina HiSeq X-TEN platform with a paired-end run of 2 × 150 bp. The quality of each read was initially verified using the software embedded in the HiSeq X-TEN sequence. The sequencing depth was more than 150×. A FASTQ file was generated for each tested sample for sequence alignment and converted to a BAM file for further analysis.

Data filtering and variant calling

Tumor-normal paired sample calling was processed during the mutation calling procedure, in order to filter out individual's private germline mutations. The resulted somatic mutations above variant allele frequency (VAF) of 2% were selected for annotation. ANNOVAR was run to screen the nonsynonymous mutations in the exonic region for further study. The candidate variants with a minor allele frequency above 0.2% as recorded in either the population database EXAC (The Exome Aggregation Consortium) v.03 or the Genome Aggregation Database (gnomAD) were filtered out for further identification of the somatic mutations.

Copy number profiling

This CNVkit v0.8.5 takes into consideration of both on- and off-target sequencing reads and applies a series of corrections for the extraneous variability sources like GC content, target footprint size and spacing to improve accuracy in copy number calling. To more precisely infer allele-specific tumor copy number profiles and mutations profiles of each sample, we used Sequenza to evaluate tumor cellularity,

ploidy and copy number states among all whole-exome sequenced sample. Briefly, it calculates GC content and processes the sequencing data from the tumor and normal specimens. It calculates the variant alleles and allelic frequency from the tumor specimen via using python script (sequenza-utils).

Phylogenetic tree construction

LICHeE reconstructs the lineage trees and sample heterogeneity by evaluating it on simulated trees of heterogeneous cancer cell lineage evolution. Briefly, identified somatic SNVs from sequencing data was firstly grouped and clustered. Then an evolutionary constraint network was constructed to capture valid evolutionary timing relationships between the mutations of each cluster pair.

Multiplex immunofluorescence staining

Protein blocking was performed using Antibody Diluent / Block (72424205, PerkinElmer) for 10 min at room temperature. Then primary Abs were incubated for 1h at 37℃ or 12 hours at 4℃. The primary Abs were grouped into two 5-antibody panels: panel 1 consisted of CD3 (ZM-0417, clone LN10; Zsbio; dilution 1:50), CD4 (ZM-0418, clone UMAB64; Zsbio; dilution 1:200), CD8 (ZA-0508, clone EP334; Zsbio; dilution 1:100), CD45 (ZM-0381, clone 2B11&PD7/26; Zsbio; dilution 1:500) and FoxP3 (ab20034, clone 236A/E7; Abcam; dilution 1:100). Panel 2 consisted of TIM3 (45208, clone D5D5R; Cell Signaling; dilution 1:400), LAG3 (ab40466, clone 17B4; Abcam; dilution 1:100), PD-1 (ZM-0381, clone UMAB199; Zsbio; dilution 1:100), PD-L1 (13684,clone E1L3N; Cell Signaling; dilution 1:100) and CD73 (13160, clone D7F9A; Cell Signaling; dilution 1:500). Next, incubation with Ploymer HRP Rb (PV-6001;Zsbio) or Ploymer HRP Ms (PV-6002;Zsbio) was performed at 37℃ for 10 min. TSA visualization was performed with the Opal seven-color IHC Kit (NEL797B001KT; PerkinElmer), which includes fluorophores DAPI, Opal 520 (CD45,CD73), Opal 570 (CD4,PD1), Opal 620 (CD3,LAG3), Opal 650 (CD8,TIM3),

Opal 690 (FOXP3,PDL1), and TSA Coumarin system (NEL703001KT; PerkinElmer). MWT was performed to remove the Ab TSA complex with Tris-EDTA buffer (pH 9). TSA single stain slides were finished with MWT and counterstained with DAPI for 5 min and were enclosed in Antifade Mounting Medium (I0052; NobleRyder).

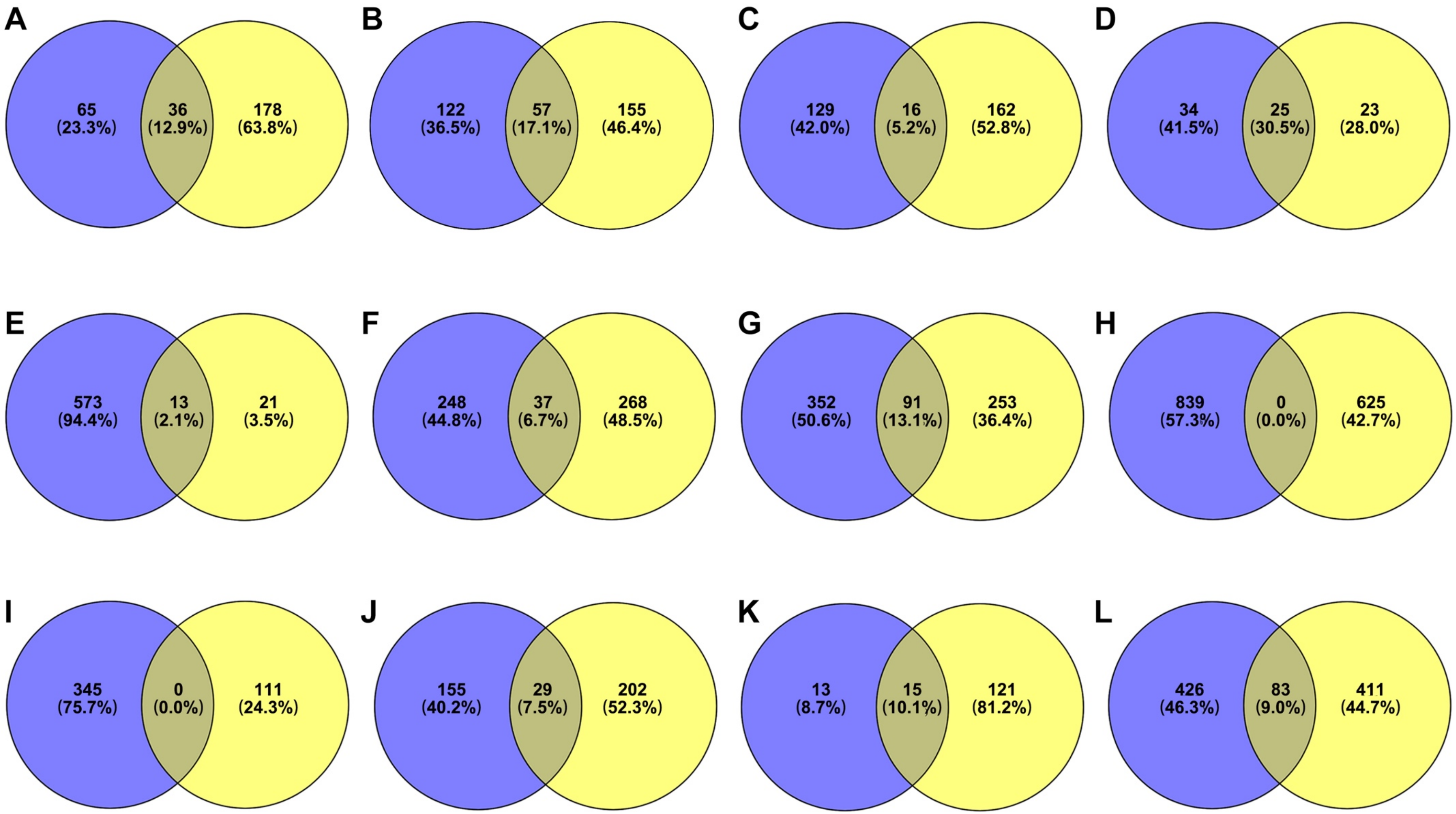
A
65 (23.3%)
36 (12.9%)
178 (63.8%)
B
122 (36.5%)
57 (17.1%)
155 (46.4%)
C
129 (42.0%)
16 (5.2%)
162 (52.8%)
D
34 (41.5%)
25 (30.5%)
23 (28.0%)
E
573 (94.4%)
13 (2.1%)
21 (3.5%)
F
248 (44.8%)
37 (6.7%)
268 (48.5%)
G
352 (50.6%)
91 (13.1%)
253 (36.4%)
H
839 (57.3%)
0 (0.0%)
625 (42.7%)
I
345 (75.7%)
0 (0.0%)
111 (24.3%)
J
155 (40.2%)
29 (7.5%)
202 (52.3%)
K
13 (8.7%)
15 (10.1%)
121 (81.2%)
L
426 (46.3%)
83 (9.0%)
411 (44.7%)

Supplementary Figure 1. Venn diagram of identified somatic mutations in each matched case. Primary lesion: yellow color; Brain metastasis: purple color. (A-L) patients No. BM31, BM32, BM33, BM34, BM35, BM36, BM37, BM38, BM39, BM40, BM42 and BM43.

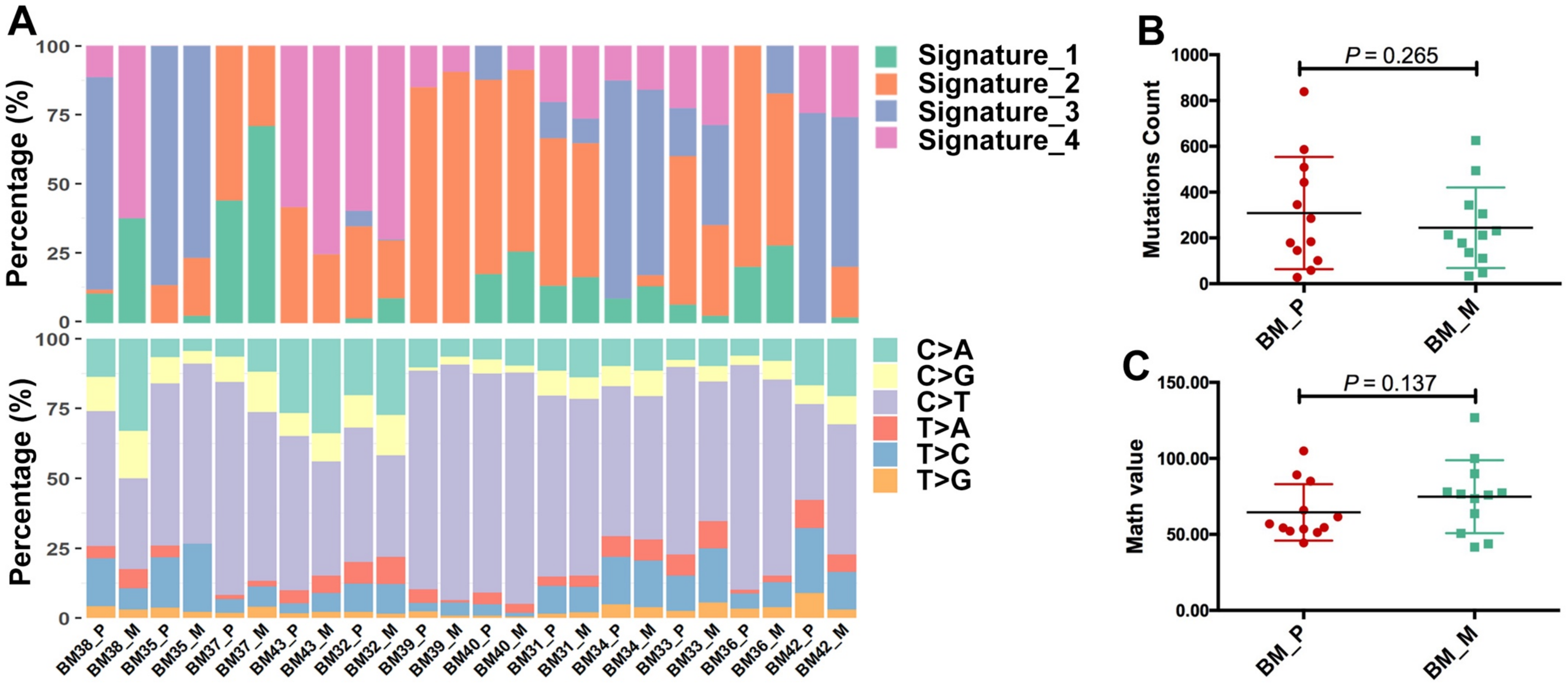

Supplementary Figure 2. Overview of mutational landscape. (A) Mutational signatures (up) and mutation fraction of the six-base substitution (down) for each sample. (B) Comparison of nonsynonymous mutation burden between primaries and BMs. (C) Comparison of math value between primaries and BMs. BM_M, brain metastatic lesion; BM_P, primary lesion.

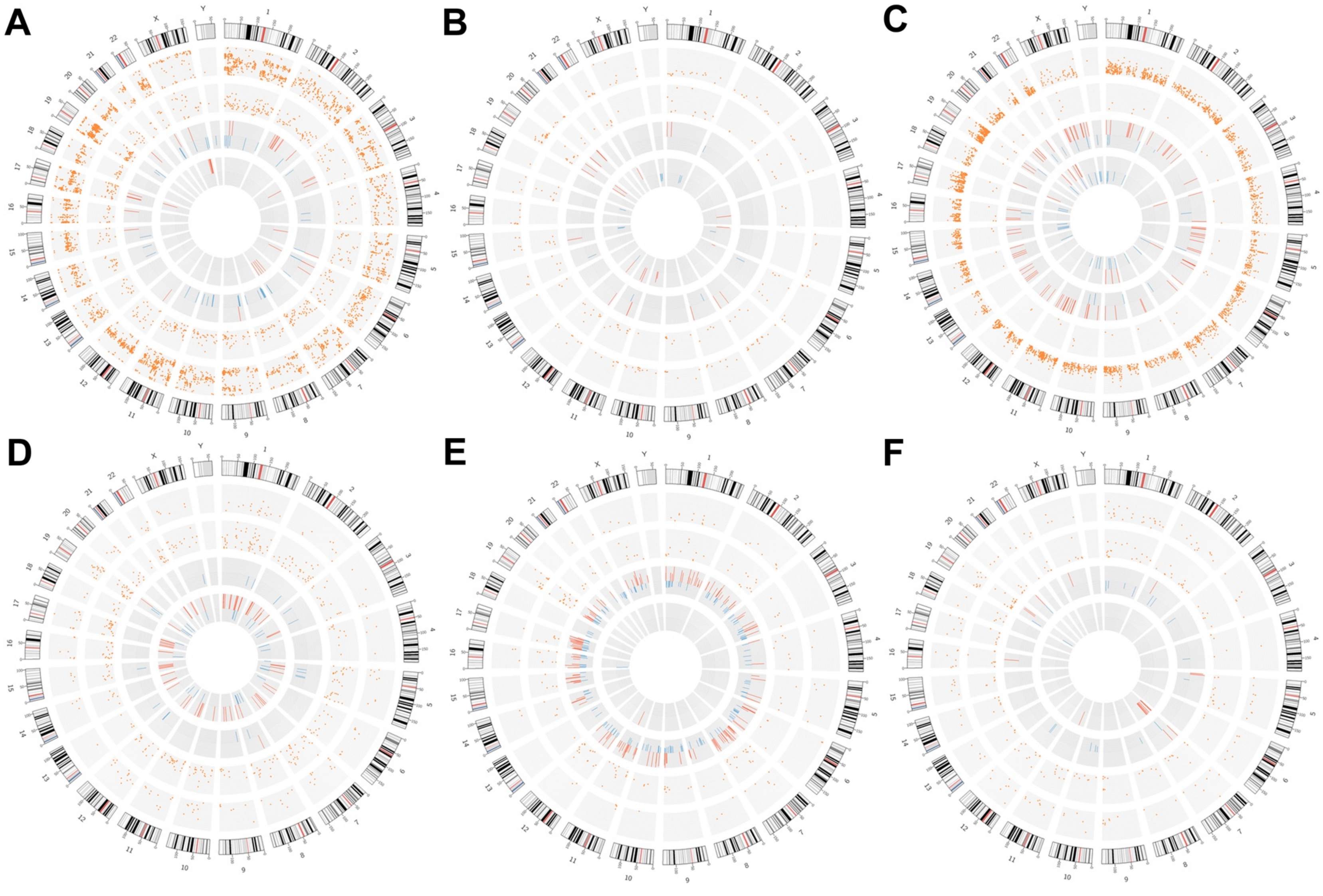
A
B
C
D
E
F

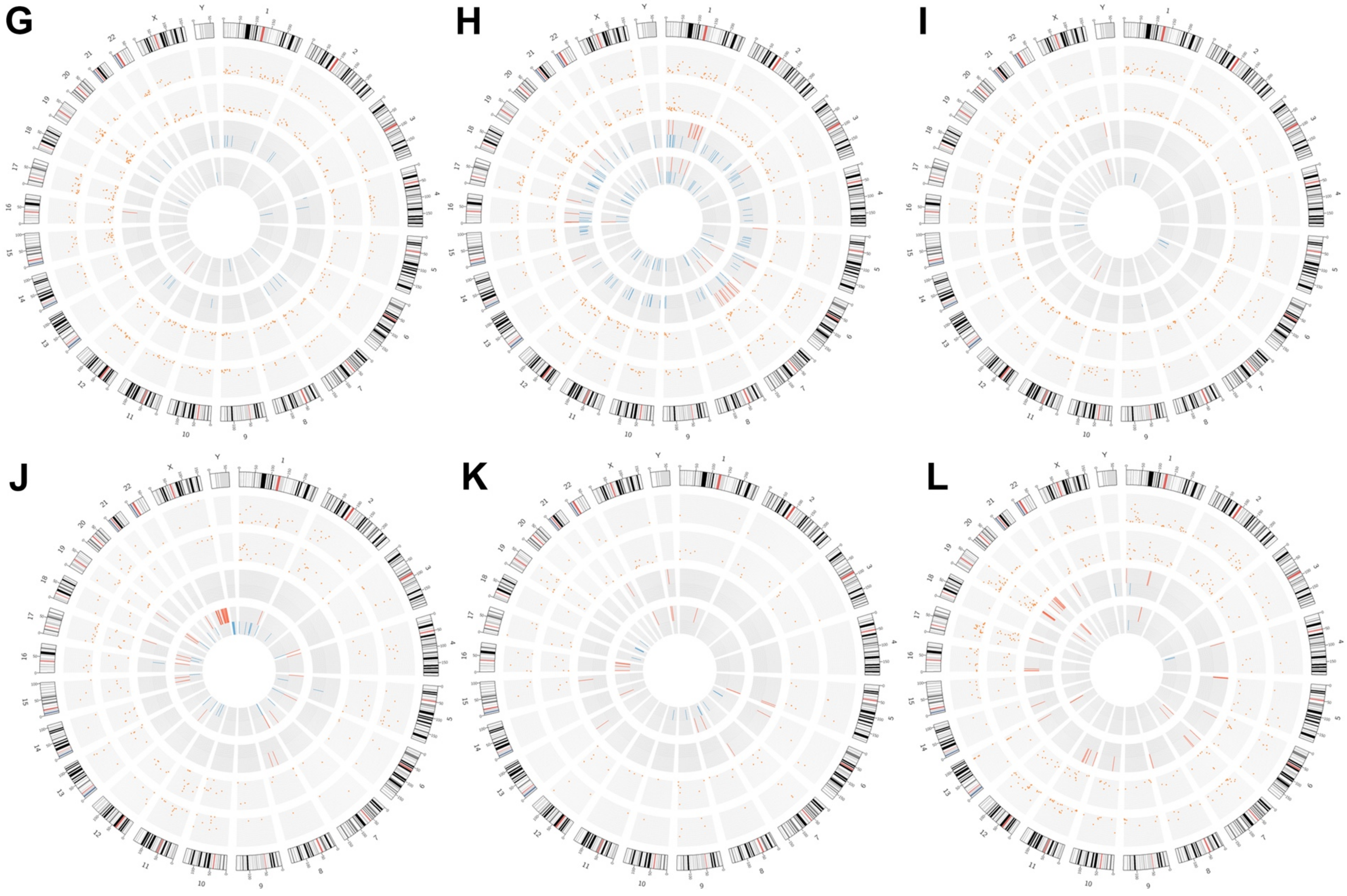
G
H
I
J
K
L

Supplementary Figure 3. Circus plot of each case. From inside to out of each circus plot: the first circle represents the CNVs of primary tumor; the second circle represents the CNVs of matched metastasis; the third circle represents the SNVs of primary tumor; the fourth circle represents the SNVs of matched metastasis. Outermost circle represents the chromosomes. (A)–(L) Circus plot of BM38, BM39, BM35, BM43, BM42, BM36, BM40, BM31, BM33, BM32, BM34 and BM37.

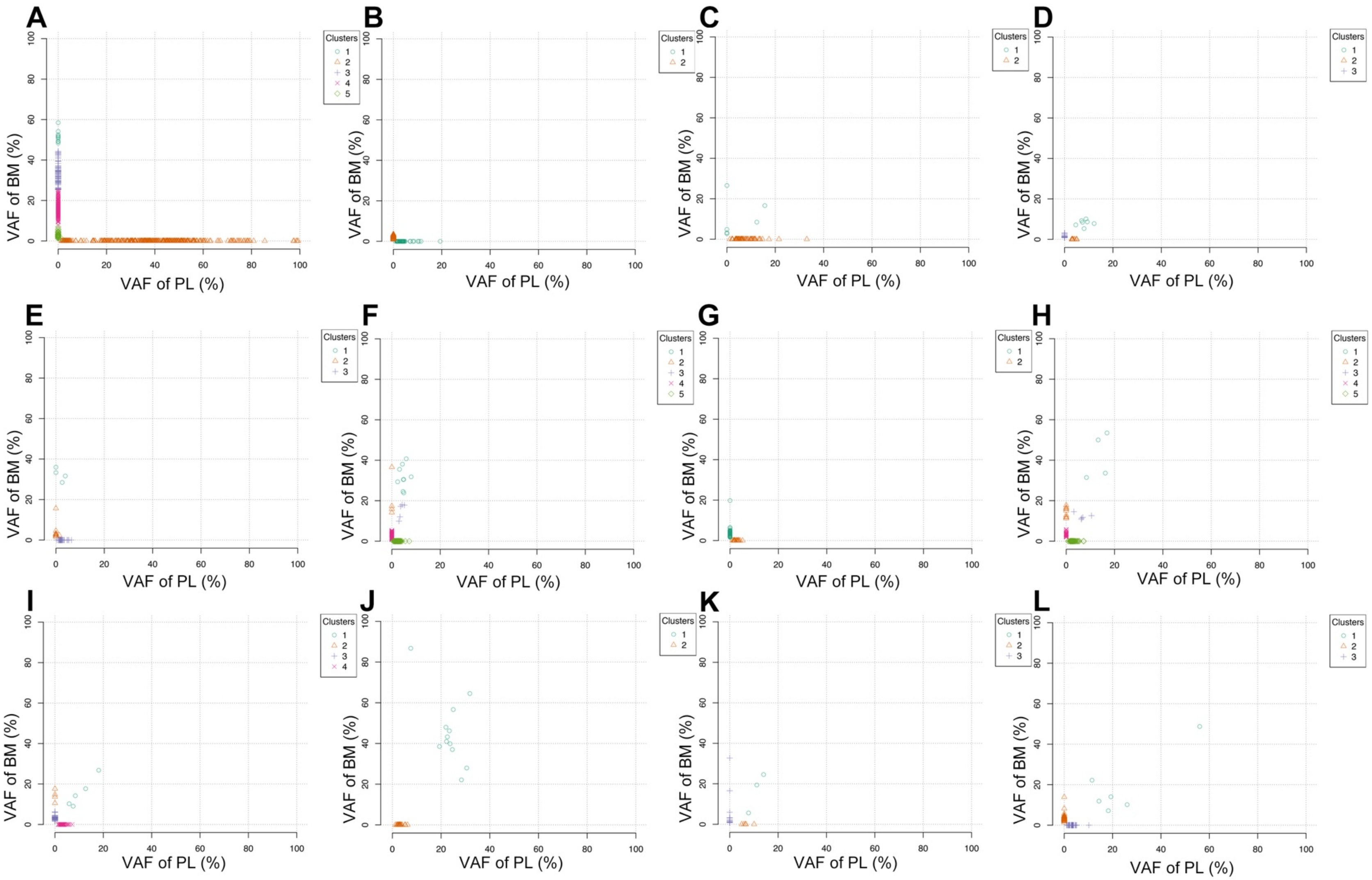

Note: VAF, variant allelic frequency; BM, brain metastasis; PL, primary lesion.

Supplementary Figure 4. Subclonal architecture of each case. (A)–(L) Subclonal architecture of BM38, BM39, BM35, BM43, BM42, BM36, BM40, BM31, BM33, BM32, BM34 and BM37. Two-dimensional scatter plots dissects the overlap clusters (variant allele frequencies of the mutations) in BM_P and BM_M within a single case. Different clusters were calculated from each tumor sample. Clusters off the axes indicate mutations in both of tumor components. Clusters on the axes reveal mutations in either BM_P or BM_M components. BM_M, brain metastatic lesion; BM_P, primary lesion.

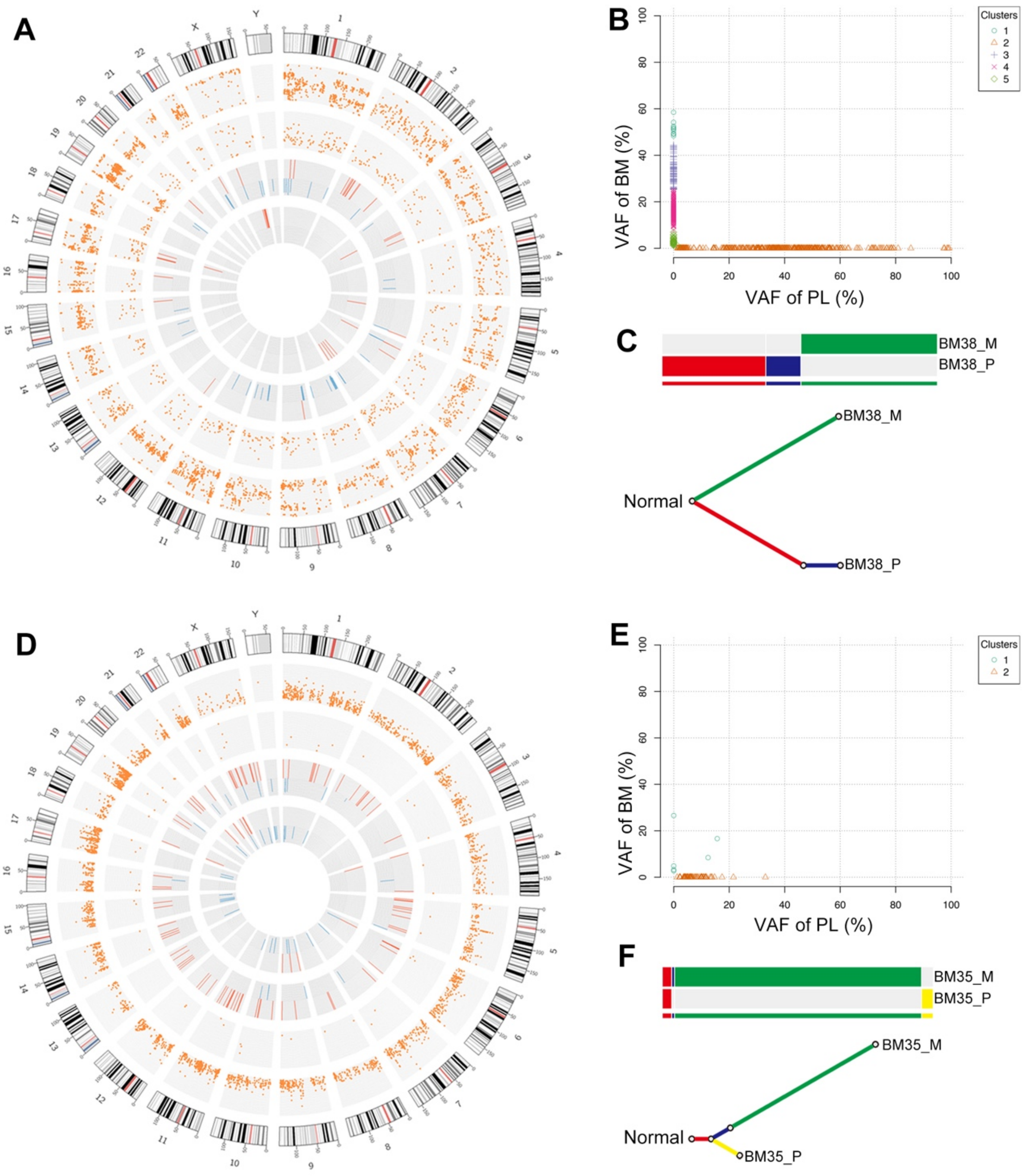

Supplementary Figure 5. Phylogenetic and subclonality analysis of two representative cases. (A) Circus plot to summarize SNVs and CNVs of BM38. (B) Subclonal architecture of BM38. (C) Phylogenetic tree of BM38. (D) Circus plot to summarize SNVs and CNVs of BM35. (E) Subclonal architecture of BM35. (F) Phylogenetic tree of BM35. BM_M, brain metastatic lesion; BM_P, primary lesion.

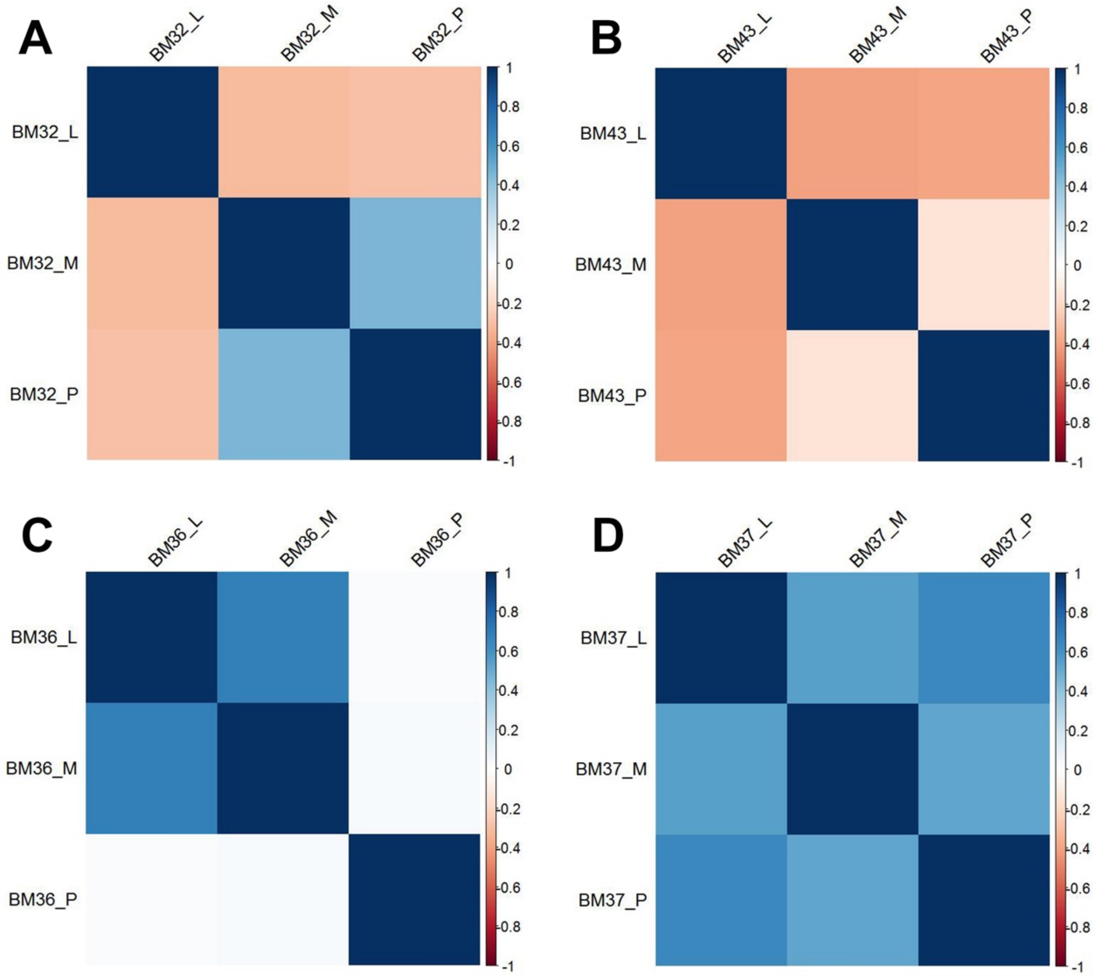

Supplementary Figure 6. Pearson Correlation Coefficients of primaries lymphatic and brain metastases in four case with synchronous lymph node metastases. (A)–(D) phylogenetic tree of BM32, BM43, BM36 and BM37. BM_P, primary lesion; BM_L, lymph node metastasis; BM_M, brain metastatic lesion.

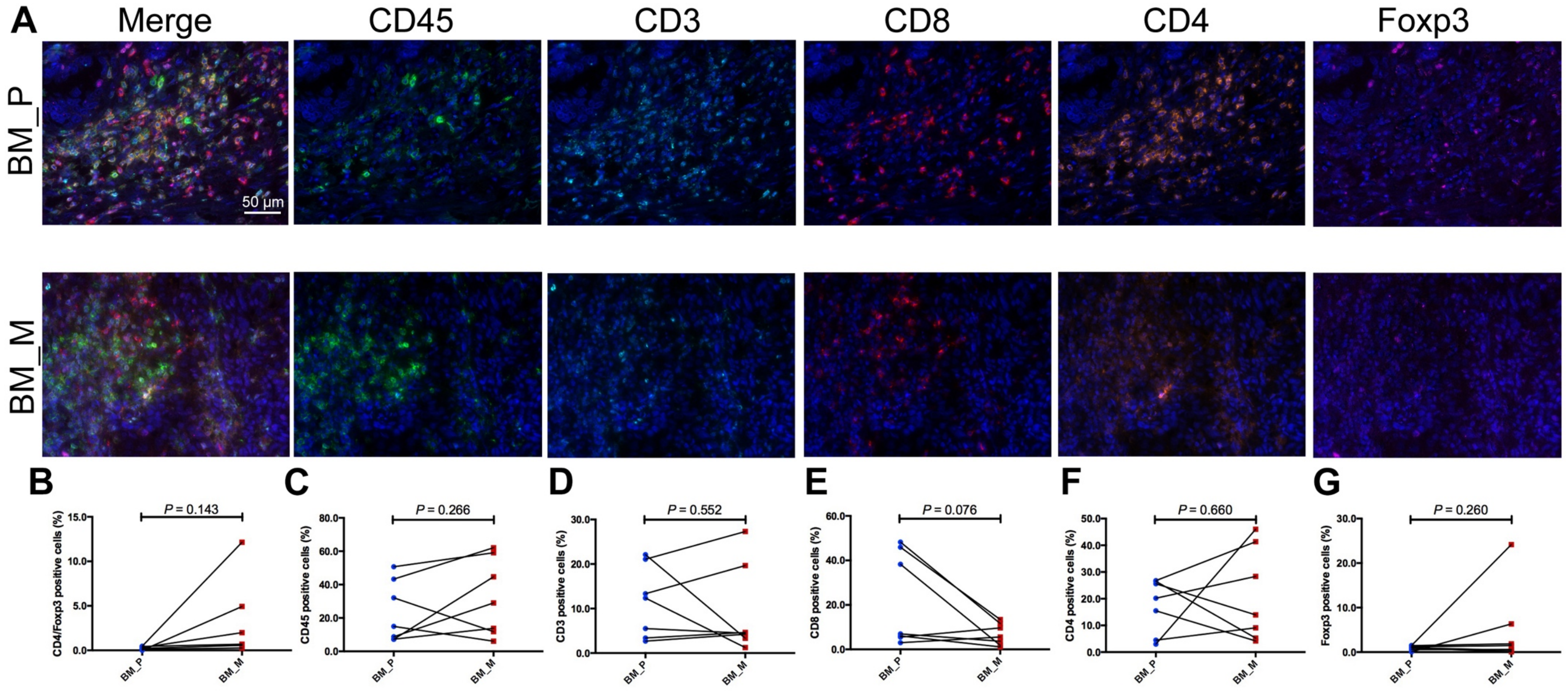

Supplementary Figure 7. MFI comparison of five immune related markers in tumors between primaries and BMs. (A) Representative images of each marker in tumor area of primary and matched brain metastasis; (B)-(G) MFI comparison of CD4$^+$Foxp3$^+$, CD45$^+$, CD3$^+$, CD4$^+$, CD8$^+$ and Foxp3$^+$. MFI, mean fluorescence intensity; BM_P, primary lesion; BM_M, brain metastatic lesion.

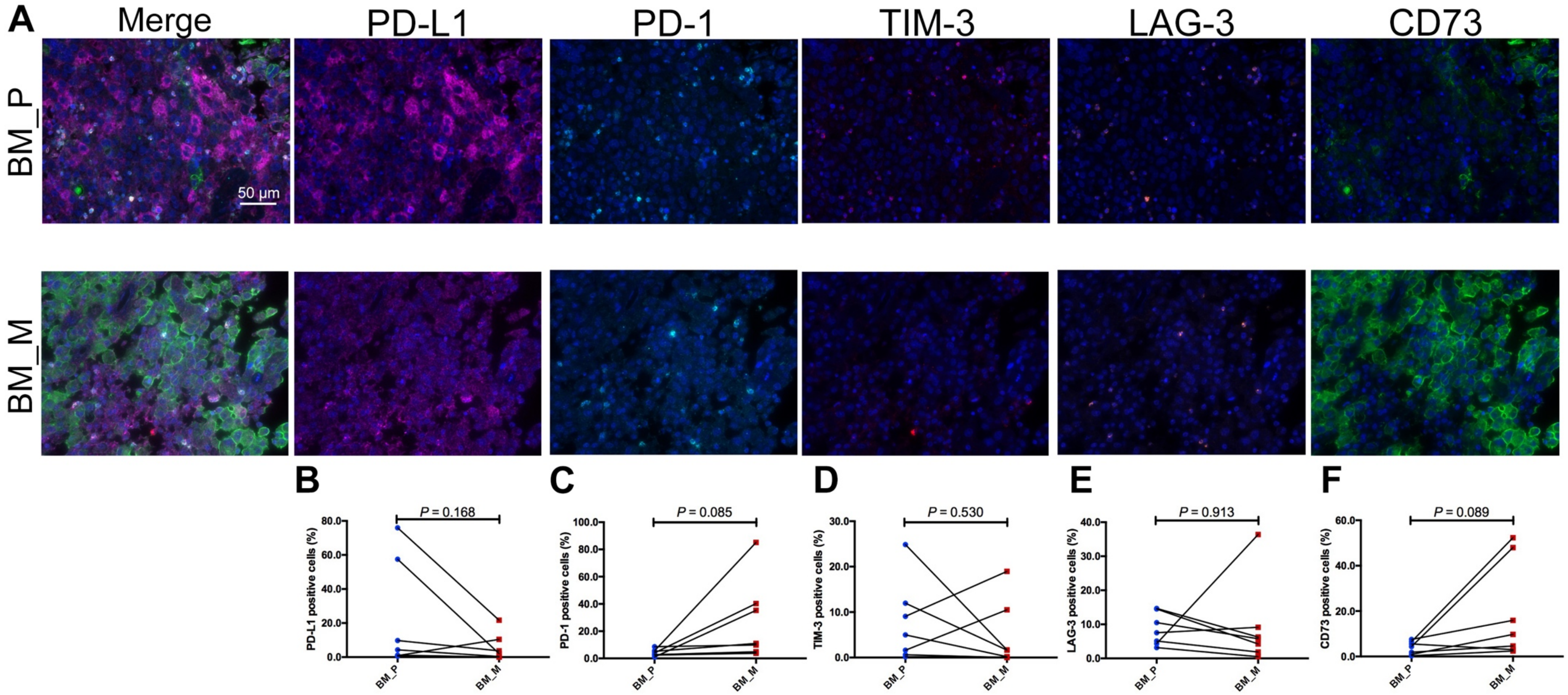

Supplementary Figure 8. MFI comparison of five immune checkpoints in tumors between primaries and BMs. (A) Representative images of each marker in tumor area of primary and matched brain metastasis; (B)-(F) MFI comparison of PD-L1, PD-1, TIM-3, LAG-3 and CD73. MFI, mean fluorescence intensity. BM_P, primary lesion; BM_M, brain metastatic lesion.

Supplementary Table 1. Baseline features of included samples.

Sample ID	Sex	Age	Smoking history	ECOG PS	Lesions	FFPE/Fresh	Pathological Purity	Cellularity	Ploidy
BM31_M	Female	46	never	1	Metastasis	FFPE	60.00%	0.45	4.3
BM31_P	Female	46	never	1	Primary	Fresh	55.00%	0.29	3.3
BM32_M	Male	53	yes	1	Metastasis	Fresh	90.00%	0.71	4.0
BM32_P	Male	53	yes	1	Primary	Fresh	60.00%	0.29	5.0
BM32_L	Male	53	yes	1	LNM	Fresh	65.00%	0.52	3.8
BM33_M	Female	50	never	1	Metastasis	FFPE	50.00%	0.36	2.8
BM33_P	Female	50	never	1	Primary	FFPE	55.00%	0.25	3.2
BM34_M	Female	47	never	1	Metastasis	FFPE	70.00%	0.51	5.6
BM34_P	Female	47	never	1	Primary	FFPE	50.00%	0.26	5.2
BM35_M	Male	36	never	1	Metastasis	FFPE	60.00%	0.36	2.5
BM35_P	Male	36	never	1	Primary	FFPE	60.00%	0.32	2.7
BM36_M	Male	62	never	1	Metastasis	Fresh	65.00%	0.40	4.3
BM36_P	Male	62	never	1	Primary	FFPE	50.00%	0.21	2.0
BM36_L	Male	62	never	1	LNM	Fresh	70.00%	0.65	2.9
BM37_M	Female	46	never	1	Metastasis	FFPE	55.00%	0.28	6.1
BM37_P	Female	46	never	1	Primary	Fresh	75.00%	0.51	3.4
BM37_L	Female	46	never	1	LNM	Fresh	80.00%	0.59	3.8
BM38_M	Male	61	yes	1	Metastasis	FFPE	65.00%	0.39	3.2
BM38_P	Male	61	yes	1	Primary	Fresh	80.00%	0.67	7.0
BM39_M	Male	54	yes	1	Metastasis	FFPE	50.00%	0.24	6.6
BM39_P	Male	54	yes	1	Primary	FFPE	50.00%	0.22	6.7
BM40_M	Female	44	never	1	Metastasis	FFPE	70.00%	0.46	2.0

BM40_P	Female	44	never	1	Primary	FFPE	55.00%	0.28	2.5
BM42_M	Female	51	never	0	Metastasis	Fresh	80.00%	0.65	2.7
BM42_P	Female	51	never	0	Primary	FFPE	50.00%	0.29	2.6
BM43_M	Male	65	never	1	Metastasis	Fresh	80.00%	0.64	2.3
BM43_P	Male	65	never	1	Primary	FFPE	50.00%	0.24	2.3
BM43_L	Male	65	never	1	LNM	Fresh	75.00%	0.41	3.2

ECOG PS, Eastern Cooperative Oncology Group performance status; LNM, lymph node metastasis; FFPE, formalin-fixed paraffin-embedded.
